# Consequences of ChemR23 Heteromerization with the Chemokine Receptors CXCR4 and CCR7

**DOI:** 10.1371/journal.pone.0058075

**Published:** 2013-02-28

**Authors:** Cédric de Poorter, Kevin Baertsoen, Vincent Lannoy, Marc Parmentier, Jean-Yves Springael

**Affiliations:** 1 Institut de Recherche Interdisciplinaire en Biologie Humaine et Moléculaire (IRIBHM) Université Libre de Bruxelles (U.L.B.), Campus Erasme, Brussels, Belgium; 2 Euroscreen SA, Gosselies, Belgium; University of York, United Kingdom

## Abstract

Recent studies have shown that heteromerization of the chemokine receptors CCR2, CCR5 and CXCR4 is associated to negative binding cooperativity. In the present study, we build on these previous results, and investigate the consequences of chemokine receptor heteromerization with ChemR23, the receptor of chemerin, a leukocyte chemoattractant protein structurally unrelated to chemokines. We show, using BRET and HTRF assays, that ChemR23 forms homomers, and provide data suggesting that ChemR23 also forms heteromers with the chemokine receptors CCR7 and CXCR4. As previously described for other chemokine receptor heteromers, negative binding cooperativity was detected between ChemR23 and chemokine receptors, i.e. the ligands of one receptor competed for the binding of a specific tracer of the other. We also showed, using mouse bone marrow-derived dendritic cells prepared from wild-type and ChemR23 knockout mice, that ChemR23-specific ligands cross-inhibited CXCL12 binding on CXCR4 in a ChemR23-dependent manner, supporting the relevance of the ChemR23/CXCR4 interaction in native leukocytes. Finally, and in contrast to the situation encountered for other previously characterized CXCR4 heteromers, we showed that the CXCR4-specific antagonist AMD3100 did not cross-inhibit chemerin binding in cells co-expressing ChemR23 and CXCR4, demonstrating that cross-regulation by AMD3100 depends on the nature of receptor partners with which CXCR4 is co-expressed.

## Introduction

Over the past decade, oligomerization has emerged as an important feature of G protein-coupled receptors (GPCRs). Heteromerization has been shown to affect some aspects of receptors function, such as their targeting to the cell surface, their pharmacology, their signalling and/or their internalization properties [1]–[3]. Among GPCR families, chemokine receptors constitute an interesting model system for studying the functional consequences of receptor heteromerization [4]; [5]. Out of the 20 chemokine receptors currently characterized, about half of them were reported to physically associate with at least one other chemokine receptor [6]–[19]. Heteromerization of chemokine receptors is potentially of crucial importance for the proper function of immune cells. With the aim of addressing this issue, we previously identified negative binding cooperativity of allosteric nature between subunits of CCR2/CCR5, CCR2/CXCR4 and CCR5/CXCR4 heteromers, i.e. the specific ligand of one receptor cross-competing for the binding of chemokines onto the others [8]–[10]. This negative binding cooperativity takes place on recombinant cell lines expressing pairs of receptors, as well as on native leukocyte populations, such as CD4^+^ T cells [8]–[10], monocytes [7] and macrophages (our unpublished results). In addition, we demonstrated that heteromerization of chemokine receptors impacts on the properties of some small molecule antagonists in vivo [7]; [9]. It is expected that functional interactions between receptors as a result of heteromerization would vary according to the cell type studied, the range of receptors expressed in these cells, their relative expression levels and their relative “affinity” for one another. In this study, we investigated the dimerization status of ChemR23, which belongs to a subfamily of G protein-coupled receptors responding to chemoattractants. Like chemokine receptors, ChemR23 is expressed by leukocyte populations such as macrophages, immature myeloid and plasmacytoid dendritic cells, as well as on a subset of NK cells [20]–[22]. Its natural ligand, chemerin, is a 137-aa protein structurally related to cathelicidin precursors, cystatins and kininogens but not to chemokines [20]. Chemerin is secreted as an inactive precursor, prochemerin, requiring proteolytic removal of six or seven amino-acids from its C-terminus to generate a potent and specific agonist of ChemR23. Activation of ChemR23 results in intracellular calcium release, inhibition of cAMP accumulation and phosphorylation of ERK-1/ERK-2 MAP kinases, through the G_i_ class of heterotrimeric G proteins. Chemerin and ChemR23 are involved in the recruitment of NK and dendritic cells into tissues in several human inflammatory diseases [20]; [21]; [23]. Accumulating data also support that chemerin and ChemR23 participate to the regulation of adipocyte metabolism [24]–[26].

In the present study, we present data indicating that ChemR23 forms homomers and heteromers with the chemokine receptors CXCR4 and CCR7 at the plasma membrane. We show that ChemR23 coexpression with chemokine receptors results in a negative binding cooperativity among the specific ligands of each receptor. Interestingly, we also show that cross-competition by the CXCR4-specific antagonist AMD3100 depends on the nature of the partner with which CXCR4 is coexpressed.

## Materials and Methods

### Ethics statement

The experiments using animals samples were carried out in strict accordance with the national, European (EU Directives 86/609/EEC) and international guidelines in use at the Université Libre de Bruxelles and in accordance with the Helsinki Declaration. All procedures were reviewed and approved by the local ethic committee (Commission d'Ethique du Bien-Etre Animal, CEBEA) of the Université Libre de Bruxelles (Permit Number: 222N and 341N). All efforts were made to minimize suffering.

### Antibodies

Antibodies used for the detection of human and mouse receptors by FACS were purchased from BD Pharmingen (anti-hCXCR4-PE, 551966; anti-hCCR7-PE, 552176 and anti-mCXCR4, 551852), Calbiochem (anti-mCCR7, 227006) or R&D Systems (anti-hChemR23, MAB362). Blocking anti-mCXCR4 used for competition binding assays was purchased from R&D Systems (MAB170).

### Cell lines and leukocyte populations

CHO-K1 cells were cultured in Ham's F12 medium supplemented with 10% fetal bovine serum (GIBCO), 100 U/ml penicillin and 100 µg/ml streptomycin (Invitrogen). Cells expressing ChemR23 and CXCR4 or ChemR23 and CCR7 were selected by 10 µg/ml G418 and 10 µg/ml blasticidin (Invitrogen). Mouse bone marrow-derived dendritic cells (BMDCs) were generated as previously described [27]. Briefly, the bone marrow was recovered by flushing femurs and cells were cultured for 14 days in RPMI 1640 containing L-glutamine (Cambrex Bioscience) supplemented with 100 U/ml penicillin, 100 µg/ml streptomycin, 50 mM 2-mercaptoethanol (Sigma), 10% heat inactivated FBS (Jacques Boy) and 20 ng/ml mouse recombinant GM-CSF (Biosource). On day 14, non adherent cells acquired a typical morphology and were collected and used as immature DCs for subsequent experiments. Surface expression of DC markers was analyzed by flow cytometry with anti-CD11c-PE (557401), anti-CD11b-FITC (557396), anti-CD40-PE (553791), anti-CD86-PE (53692, all from BD Pharmingen), showing around 90% cell purity.

### BRET assays

The cDNAs encoding EYFP and a humanized form of Renilla luciferase were fused in frame to the 3′ end of ChemR23, CXCR4 and CCR7 cDNAs in the pcDNA3.1 vector. Human embryonic kidney cells (HEK-293T) were transfected by the calcium phosphate precipitation method, using a constant amount of plasmid DNA but various ratios of plasmids encoding the fusion protein partners. Mock-transfected cells were used as control in order to subtract raw basal luminescence and fluorescence from the data. Expression of EYFP fusion proteins was measured by recording fluorescence at 535 nm following excitation at 485 nm, using a Mithras LB940 Multilabel Reader (Berthold). Expression of RLuc fusion proteins was measured by recording the luminescence of the cells after incubation with 2.5 µM coelenterazine H (Promega). In parallel, BRET was measured as the fluorescence of the cells at 535 nm at the same time points. The BRET ratio is defined as [(emission at 510–590)/(emission at 440–500)] – Cf where Cf corresponds to (emission at 510–590)/(emission at 440–500) for the hRLuc construct expressed alone in the same experiment.

### HTRF assays

HEK-293T were transfected by the calcium phosphate precipitation method with plasmids encoding HA-ChemR23 with or without various receptor constructs (see Results). After 48 h, cells were harvested and incubated in PBS containing 0.5 µg/ml of Europium (Eu)-labelled anti-HA (FRET Energy donor) and 5 µg/ml of XL665-labelled anti-HA antibody (FRET Energy acceptor) (CisBio Bioassays). After one hour incubation at 37°C, the FRET ratio (emission at 665 nm/emission at 620 nm) is measured after an excitation pulse of the Europium at 330 nm on a dual-wavelength time resolved fluorimeter (RubyStar, Isogen). The specific signal ΔF is defined as (FRET ratio – pSVL)/(pSVL) where pSVL corresponds to the FRET ratio for mock transfected cells in the same experiment.

### Binding assays

Competition binding experiments were performed as previously described [8]. Membrane preparations were incubated in the assay buffer (50 mM Hepes pH 7.4, 1 mM CaCl_2_, 5 mM MgCl_2_, 0.5% BSA) with 0,1 nM ^125^I-chemerin, 0,1 nM ^125^I-CCL19 or 0.1 nM ^125^I-CXCL12 as tracers and variable concentrations of unlabeled competitors. Samples were incubated for one hour and bound tracer was separated by filtration through GF/B filters presoaked in 1% BSA. Filters were counted in a γ-scintillation counter. Binding parameters were determined with the PRISM software (Graphpad Softwares) using nonlinear regression applied to single site or two sites binding models. The software compared the sum-of-square and the degree of freedom of each regression by using the F test and selected the most appropriate equation.

### Intracellular calcium mobilization assay

Functional responses were analyzed with an aequorin-based assay as described [8]. Cells were incubated for 4 h in the dark in the presence of 5 µM coelenterazine H (Promega Corporation), then diluted 5-fold before use. The cell suspension (25,000 cells/well) was added to wells containing various concentrations of agonists and luminescence was measured for 30 sec in an EG&G Berthold luminometer (PerkinElmer Life Sciences). Half-maximal effective concentrations (EC_50_) were determined with the PRISM software using nonlinear regression applied to a sigmoidal dose-response model.

### Internalization assay

Adherent CHO-K1 cells stably expressing the receptors were incubated with 100 nM of ligand at 37°C. After 90 minutes, the reaction was stopped by replacing the medium with ice-cold PBS. Surface-bound chemokines and chemerin were removed by an acid wash step (50 mM glycine HCl buffer, pH 2.7, containing 150 mM NaCl). Cells were subsequently washed three times with ice-cold PBS before FACS analysis. Surface expression of receptors after ligand incubation is expressed as percentage of expression compared to untreated cells (100%).

## Results

### Heteromerization of ChemR23 with CXCR4 and CCR7

**Figure 1:**
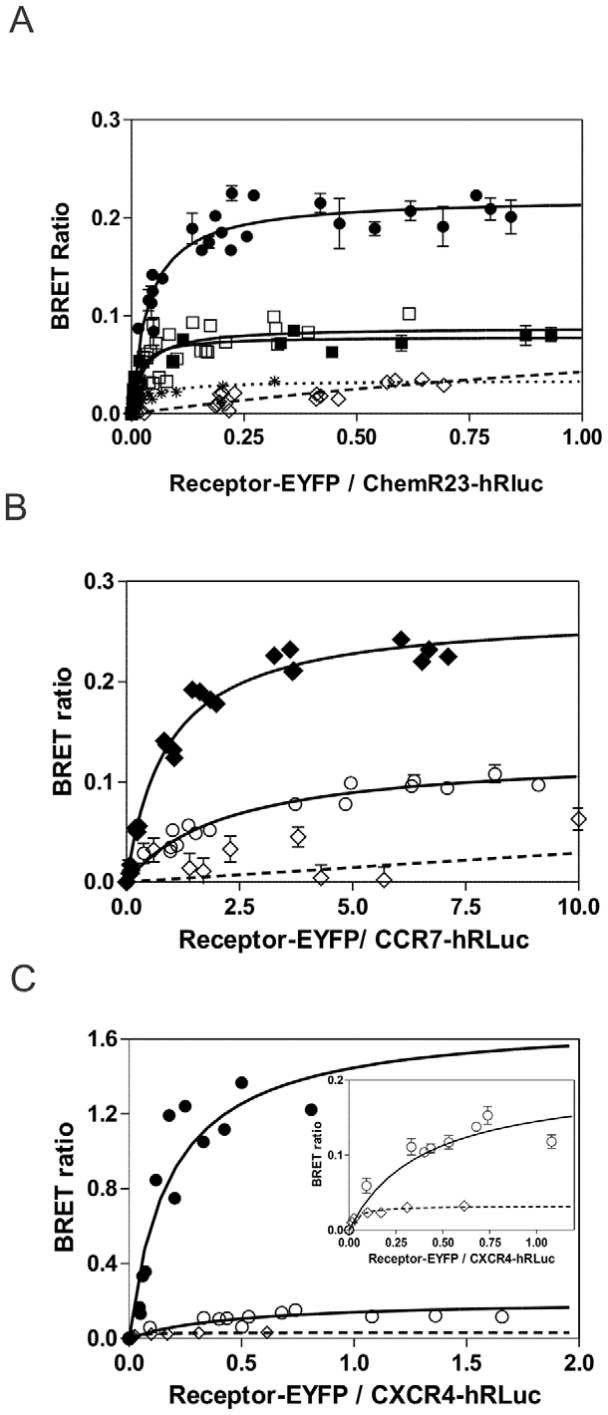
Homo- and heteromerization of ChemR23 as measured by BRET. [**A**] HEK293T cells were transfected with a constant amount of the ChemR23-h*R*Luc construct and increasing amounts of the ChemR23-EYFP (•), CXCR4-EYFP (▪) or CCR7-EYFP (□) constructs. As a control, increasing amounts of GABAbR2-EYFP (⋄, dotted curve) or TSHR-EYFP (*, dotted curve) were used as BRET acceptors [**B–C**] HEK293T cells were transfected with a constant amount of the CCR7-h*R*Luc or CXCR4-h*R*Luc constructs and increasing amounts of CCR7-EYFP (**♦**), CXCR4-EYFP (•) or ChemR23-EYFP (○) constructs. As a control, increasing amounts of GABAbR2-EYFP (⋄, dotted curve) was used as BRET acceptor. The BRET signal was recorded 5 minutes after addition of coelenterazine H. All data points were performed in triplicate (error bars indicate S.E.M.).

We have previously shown that heteromerization of chemokine receptors is associated with negative binding cooperativity across their respective ligands [7]–[10]. In the present study, we investigated the ability of chemokine receptors to interact with ChemR23, a member of the chemoattractant receptor family [20], and tested whether negative binding cooperativity could apply as well for heteromers involving ChemR23. In a first step, we investigated the extent of ChemR23 heteromerization in HEK293T cells by using the bioluminescence resonance energy transfer (BRET) assay. An energy transfer was detected between ChemR23-hRLuc and ChemR23-EYFP as well as between ChemR23-hRLuc and CXCR4-EYFP or CCR7-EYFP. As a specificity control, the GABAbR2-EYFP and TSHR-EYFP were used, which led to a much lower energy transfer (Figure 1 A). The calculated BRET_50_ parameters were in the same range for homo- and heteromers but the BRET_MAX_ values were about two-fold lower for ChemR23 heteromers as compared to ChemR23 homomers (Figure S1 A). BRET was also detected between CXCR4-hRLuc and ChemR23-EYFP and between CCR7-hRLuc and ChemR23-EYFP (Figure 1 B, C). Again, the BRET_MAX_ values were two to height fold lower than those obtained for the respective CXCR4 and CCR7 homomers and a two-fold increase of the BRET_50_ values was also detected for each heteromer. Nevertheless, the energy transfer in heteromers was much higher than when GABAbR2-EYFP was used as a negative control (Figure 1 B and 1 C inlet). Not surprisingly, no BRET signal was detected between GABAbR1-Rluc and ChemR23-EYFP, CCR7-EYFP or CXCR4-EYFP (Figure S1 A). The lower BRET_MAX_ values detected for heteromers reflect most likely a less favorable relative orientation of BRET donor and acceptor moieties. The variations of BRET_50_ values between homo- and heteromers were dependent upon which receptor of the pair was the BRET donor, which prevents any definitive conclusion regarding the relative propensity of receptors to interact.

**Figure 2:**
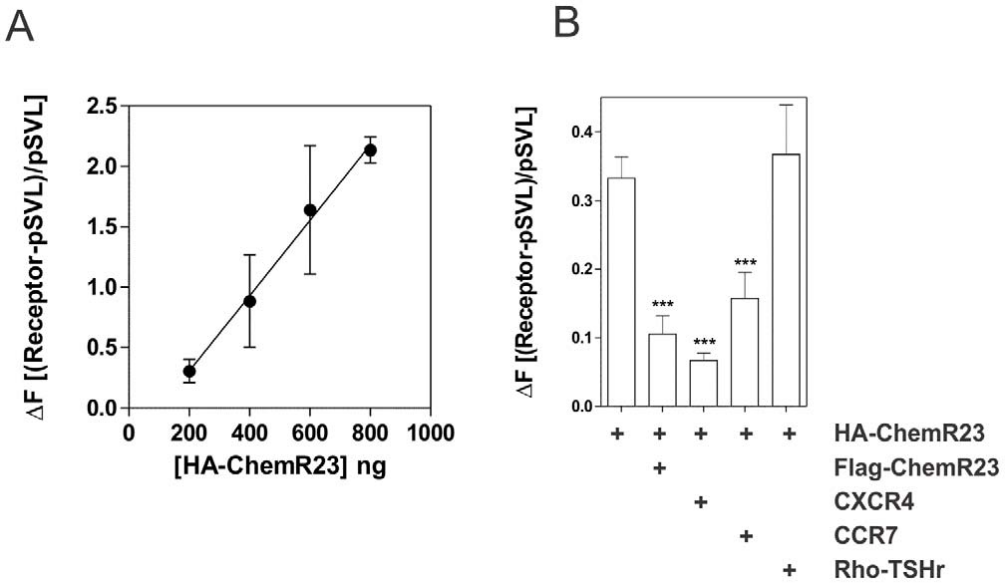
Homo- and heteromerization of ChemR23 as measured by HTRF. [**A**] HEK293T cells were transfected with increasing amounts of HA-ChemR23 and incubated for one hour with (Eu)-labelled anti-HA and XL665-labelled anti-HA (ratio 1∶10) and the homogenous FRET ratio measured. [**B**] HEK293T cells were transfected with HA-ChemR23 only or with HA-ChemR23 and Flag-ChemR23, CXCR4, CCR7 or Rho-tagged TSHR used as competitors. Cells were incubated with (Eu)-labelled anti-HA and XL665-labelled anti-HA (ratio 1∶10) and the homogenous FRET ratio measured. For each transfection, the expression level of HA-ChemR23 and competitors was measured by FACS (Figure S1 B). All data points were performed in duplicate (error bars indicate S.E.M.).

With the aim of exploring further the oligomerization status of ChemR23 at the plasma membrane of HEK293T cells, we relied on a Homogenous Time Resolved FRET (HTRF) assay. Cells expressing variable amounts of HA-tagged ChemR23 were incubated with a constant 1∶10 ratio of energy-donor and -acceptor antibodies and the FRET signal resulting from the proximity of antibody pairs was recorded (Figure 2 A). In our experiments, the acceptor and donor compete for the same binding site (HA-tag). As more receptors are expressed, the amount of receptor-bound donor and acceptor increases as well, thereby increasing the FRET signal [35]. However, this increasing FRET signal might reflect either specific interactions or random contacts due to receptor crowding in the plasma membrane. With the aim of investigating the specificity of receptor interactions, competition experiments were performed by co-transfecting various constructs in HA-ChemR23-expressing cells. Results showed that the FRET signal between the (Eu)-tagged and the XL668-tagged anti-HA antibodies decreased strongly when cells co-expressed Flag-tagged ChemR23 or untagged CXCR4 or CCR7, suggesting that these receptors interact with HA-ChemR23 and compete for ChemR23 homomerization (Figure 2 B). In contrast, no competition was detected when cells were cotransfected with the Rho-tagged TSH receptor used as a negative control. As an additional control, we showed that the drop of FRET signal was not due to a decrease in HA-ChemR23 level, as the amount of HA-ChemR23 at the cell surface, as measured by FACS, remained similar whether the cells expressed or not the competitors (Figure 2 B and Figure S1 B). Altogether, the HTRF results support the BRET data and indicated that ChemR23 is present at the plasma membrane as homo- and heteromers.

### Construction and characterization of cell lines co-expressing ChemR23 and CXCR4 or CCR7

In order to study the consequences of ChemR23 heteromerization, CHO-K1 cell lines stably co-expressing ChemR23 with CXCR4 or CCR7 were generated and analyzed for the level of expression of each receptor by FACS and saturation binding assays (Figure S2). In saturation binding assays, the B_MAX_ values of clone 10, co-expressing ChemR23 and CXCR4, were estimated to 1.53±0.17 pmoles/mg membrane proteins for ChemR23, and to 3.21±0.05 pmoles/mg membrane proteins for CXCR4. The B_MAX_ values of clone 26, co-expressing ChemR23 and CCR7, were estimated to 1.76±0.12 pmoles/mg membrane proteins for ChemR23, and to 4.00±0.10 pmoles/mg membrane proteins for CCR7. These B_MAX_ values are similar to those of parental cell lines expressing one receptor only, demonstrating that co-expression of ChemR23 with CCR7 or CXCR4 did not modify the apparent number of these receptors. In addition, FACS analysis showed that the selected clones were homogeneous in terms of receptor expression and regular testing confirmed the stability of this expression over time.

### Binding and functional properties of cells co-expressing ChemR23 and CXCR4 or CCR7

**Figure 3:**
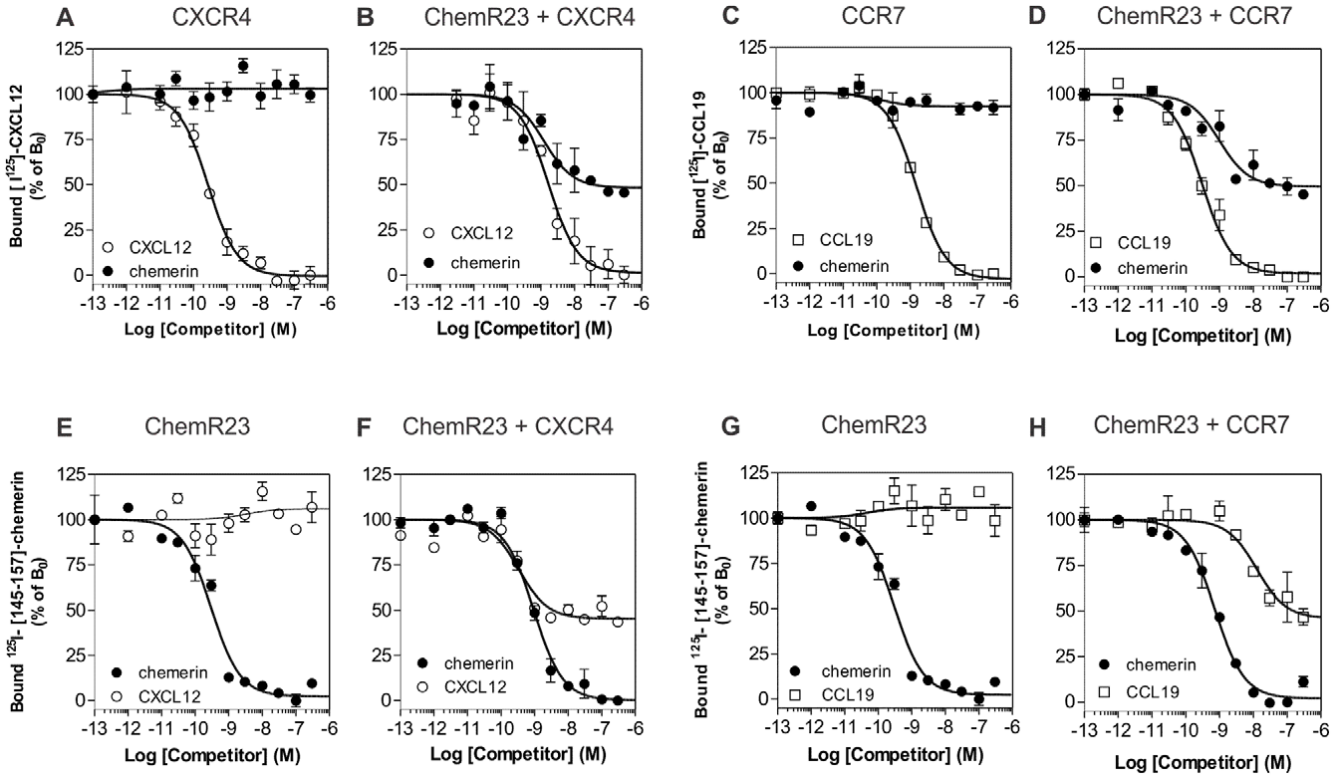
Competition binding assays in cells co-expressing ChemR23 and CCR7 or CXCR4. Competition binding assays were performed on cells expressing CXCR4 (A), CCR7 (C) or ChemR23 only (E and G), and on cells co-expressing ChemR23 and CXCR4 (B and F), or ChemR23 and CCR7 (D and H). Cells were incubated with 0.1 nM ^125^I-CXCL12 (A and B), ^125^I-CCL19 (C and D) or ^125^I-[145–157]-chemerin (E–H), as tracers and increasing concentrations of unlabelled chemerin (•), CCL19 (□) or CXCL12 (○) as competitors. After one hour incubation, unbound tracers were separated by filtration and filters washed twice before counting. The data were normalized for nonspecific binding (0%) in the presence of 300 nM of competitor, and specific binding in the absence of competitor (100%). All points were run in triplicates (error bars indicate S.E.M.). The displayed data are representative of two independent experiments.

We next compared the ability of ligands of the three receptors to inhibit the binding of specific radiolabelled tracers (^125^I-chemerin, ^125^I-CXCL12 or ^125^I-CCL19) to cells expressing single receptors or pairs of receptors. Competition binding assays showed that unlabelled chemerin inhibits the binding of ^125^I-CXCL12 or ^125^I-CCL19 on cells co-expressing ChemR23 and CXCR4 (Figure 3 B), or ChemR23 and CCR7 (Figure 3 D), but not on cells expressing CXCR4 only (Figure 3 A) or CCR7 only (Figure 3 C). Conversely, the CXCR4- and CCR7-specific ligands, CXCL12 and CCL19 respectively, inhibited the binding of ^125^I-chemerin, only on cells co-expressing ChemR23 and one of the chemokine receptors (Figure 3 F and 3 H). The IC_50_ values calculated for heterologous competition were in the same range as those estimated for homologous competition on cells expressing single receptors (Table 1). These results show thus a negative binding cooperativity between the binding pockets of ChemR23/CXCR4 and ChemR23/CCR7, similar to what we reported previously for chemokine receptor pairs. To our knowledge, this is the first example of negative binding cooperativity involving chemokines and a structurally unrelated chemoattractant protein.

**Table 1:** Binding parameters of CHO-K1 cells expressing ChemR23, CXCR4 and/or CCR7.

Cells	Tracer	Competitor	IC_50_ (nM)	% of inhibition
ChemR23	[^125^I]-[145–157]-chemerin	Chemerin	0,56±0,13	100
CCR7	[^125^I]-CCL19	CCL19	2,42±0,86	100
CXCR4	[^125^I]-CXCL12	CXCL12	0.33±0.07	100
ChemR23 + CCR7	[^125^I]-CCL19	CCL19	0,27±0,05	100
		Chemerin	0,69±0,30	49,5±1,1
	[^125^I]-[145–157]-chemerin	Chemerin	0,77±0,18	100
		CCL19	6,85±4,56	55,5±1,6
ChemR23+ CXCR4	[^125^I]-CXCL12	CXCL12	0.76±0.65	100
		Chemerin	0,81±0,28	48,2±4,2
	[^125^I]-[145–157]-chemerin	Chemerin	0,63±0,12	100
		CXCL12	0,46±0,09	43,4±7,1

Binding parameters were measured on CHO-K1 cells expressing ChemR23, CXCR4, CCR7 or combinations of these receptors. The IC_50_ and % of inhibition values were obtained from competition binding experiments as displayed in Figure 3. Values represent the mean ± S.E.M. of at least three independent experiments.

**Figure 4:**
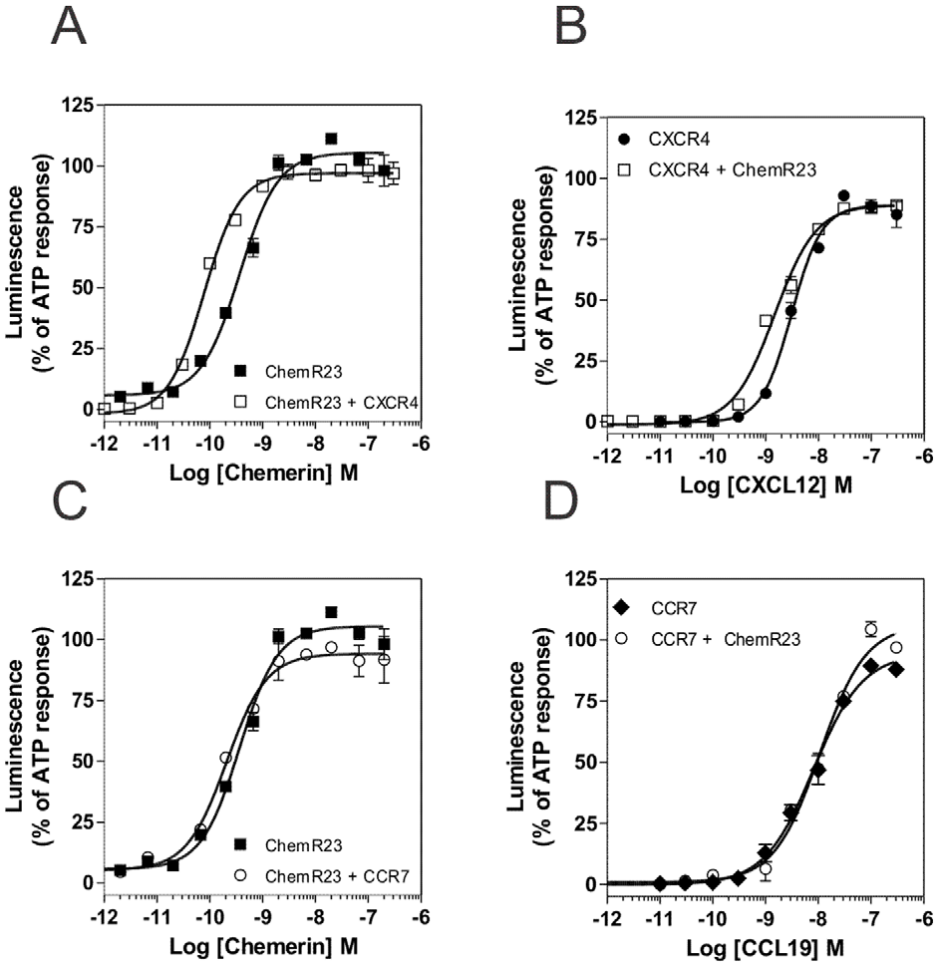
Aequorin-based functional assay in cells co-expressing ChemR23 with CCR7 or CXCR4. The functional responses of CHO-K1 cells expressing ChemR23 (▪), CXCR4 (•), CCR7 (♦), ChemR23 + CXCR4 (□) or ChemR23 + CCR7 (○) were measured using the aequorin-based calcium mobilization assay. Cells pre-loaded with coelenterazine H were stimulated with increasing concentrations of chemerin (**A, C**), CXCL12 (**B**) or CCL19 (**D**) and luminescence was recorded for 30 s. The results were normalized for baseline activity (0%) and the maximal response obtained with 25 µM ATP (100%). All points were run in triplicates (error bars indicate S.E.M.). The displayed data are representative of three independent experiments.

We then compared the functional response of cells co-expressing CXCR4 and ChemR23 or CCR7 and ChemR23 to cell lines expressing only one receptor. The concentration-action curves upon stimulation by chemerin were similar whatever ChemR23 was expressed alone or with one of the chemokine receptors (Figure 4 A and 4 C, Table 2). Similarly, concentration-action curves upon stimulation by CXCL12 or CCL19 were not altered by the presence of ChemR23 (Figure 4 C and 4 D, Table 2).

**Table 2:** Concentration-dependent agonist-induced calcium mobilization in CHO-K1 cells expressing ChemR23, CXCR4 and/or CCR7.

Cells	Agonist	EC_50_ (nM)	E_MAX_ (% of ATP response)
ChemR23	Chemerin	0,31±0,04	101±1
CCR7	CCL19	7,66±2,99	99±7
CXCR4	CXCL12	2,21±0,22	94±14
ChemR23 + CCR7	CCL19	4,18±3,90	100±1
	Chemerin	0,37±0,14	92±3
ChemR23+ CXCR4	CXCL12	1,80±0,30	84±14
	Chemerin	0,10±0,10	101±5

Functional parameters were measured on CHO-K1 cells expressing ChemR23, CXCR4, CCR7 or combinations of these receptors. The EC_50_ and E_MAX_ values were obtained from dose response experiments as displayed in Figure 4. Values represent the mean ± S.E.M. of at least three independent experiments.

**Figure 5:**
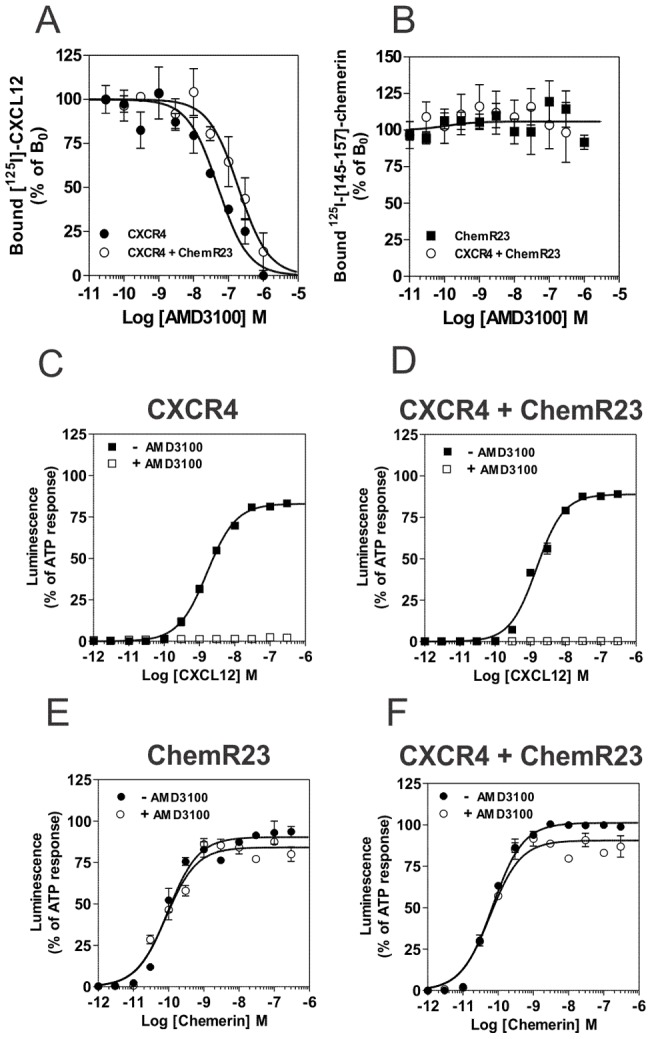
Effect of AMD3100 on binding and functional properties of cells expressing CXCR4 and ChemR23. [**A, B**] Competition binding assays were performed on cells expressing CXCR4 (•) and ChemR23 (▪) only, or cells co-expressing CXCR4 and ChemR23 (○). Cells were incubated with 0.1 nM ^125^I-CXCL12 (**A**) or ^125^I-[145–157]-chemerin (**B**), as tracers and increasing concentrations of unlabelled AMD3100 as competitor. After one hour incubation, unbound tracers were separated by filtration and filters washed twice before counting. The data were normalized for nonspecific binding (0%) in the presence of 300 nM of unlabelled CXCL12 or chemerin respectively, and specific binding in the absence of competitor (100%). All points were run in duplicate (error bars indicate S.E.M.). The displayed data are representative of two independent experiments**.** The functional responses of CHO-K1 cells expressing CXCR4 (**C**), ChemR23 (**E**) or both receptors (**D, F**) were measured by using the aequorin-based calcium mobilization assay. [**C, D**] Cells preloaded with coelenterazine were stimulated with increasing concentrations of CXCL12 (▪) or CXCL12 + AMD3100 (□). [**E, F**] Cells preloaded with coelenterazine were stimulated with increasing concentration of chemerin (•) or chemerin + AMD3100 (○). The results were normalized for baseline activity (0%) and the maximal response obtained with 25 µM ATP (100%). All points were run in triplicates (error bars indicate S.E.M.). The displayed data are representative of three independent experiments.

In previous studies, we also showed that the CXCR4-specific antagonist AMD3100 inhibits the binding of CCR2- and CCR5-specific ligands, as the result of CXCR4 heteromerization with CCR2 and CCR5 [7]; [9]. We investigated therefore whether AMD3100 also inhibits the binding of chemerin in cells co-expressing CXCR4 and ChemR23 (Figure 5 A and 5 B). In contrast to what we reported previously for other CXCR4 heteromers, AMD3100 did not inhibit the binding of chemerin on cells co-expressing ChemR3 and CXCR4, indicating that cross-competition by AMD3100 depends on the nature of the receptor with which CXCR4 is co-expressed. We also tested the effect of the CXCR4-specific antagonist AMD3100 on the functional response of cells co-expressing CXCR4 and ChemR23, and showed, in agreement with our binding data, that AMD3100 antagonized the functional response of CXCR4 but did not inhibit the response to chemerin (Figure 5 C–F). Finally, we tested whether ChemR23 heteromerization impacts on receptor endocytosis (Figure S3). Cells co-expressing ChemR23 and CXCR4 or ChemR23 and CCR7 were stimulated with 100 nM chemerin for 90 minutes. After removal of bound chemerin by an acid wash step, the amount of cell surface receptors was estimated by FACS. Results showed that stimulation with chemerin induced a decrease of cell surface ChemR23 but not of CXCR4 or CCR7. Conversely, stimulation of CCR7 or CXCR4 with either CCL19 or CXCL12 induced internalization of the chemokine receptors but without decrease of cell surface ChemR23, suggesting thus that ChemR23 does not co-internalized with chemokine receptors.

### Relevance of ChemR23/CXCR4 heteromers in native cells

**Figure 6:**
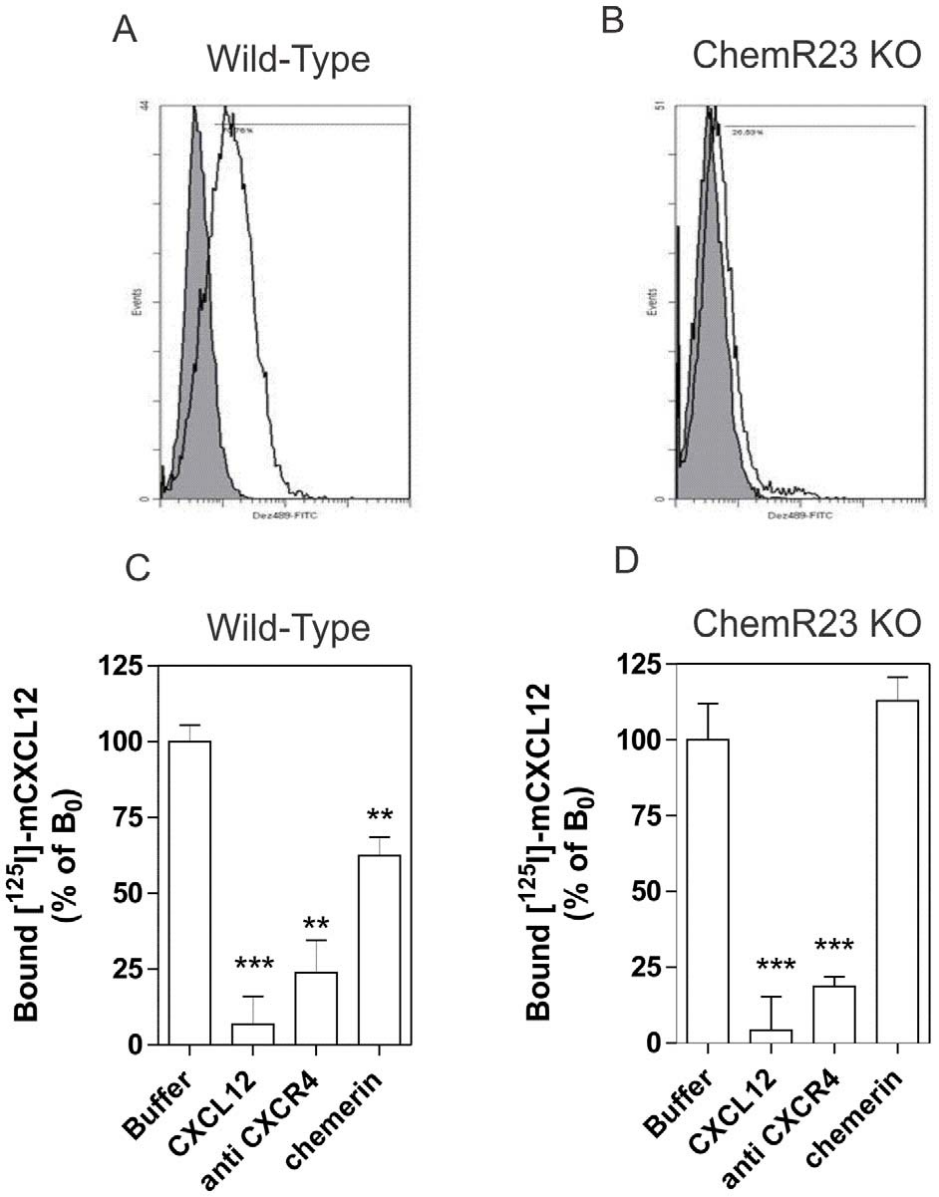
Competition binding assays on BMDCs. [**A, B**] FACS analysis showing the cell surface expression of mChemR23/Dez on BMDCs generated from wild-type or ChemR23^−/−^ mice. Cells were incubated with an anti-mChemR23 antibody (open histogram) or a control isotype (filled histogram). [**C, D**] Competition binding assays were performed on BMDCs generated from wild-type (**C**) or ChemR23^−/−^ mice (**D**). Purified cells were incubated with 0.2 nM ^125^I-CXCL12 as tracer, and CXCL12 (300 nM), chemerin (300 nM) or a monoclonal anti-CXCR4 antibody (10 µg/ml) as competitors. After one hour incubation, unbound tracer was separated by filtration and filters washed twice before counting. The data were normalized for non-specific binding (0%) and specific binding in the absence of competitor (100%). Statistical significance as compared to the 100% values was tested by two-way analysis of variance followed by Tukey's test (***, *P*<0.001; **, P<0.01). All data points were performed in triplicate and the displayed data are the mean of five experiments performed with three independent cell preparations (error bars indicate S.E.M.).

Finally, we tested whether negative binding cooperativity could be detected in cells co-expressing ChemR23 and CXCR4 endogenously, using bone marrow dendritic cells (BMDC) generated from wild-type C57/Bl6 mice. Specific ^125^I-CXCL12 binding was detected on BMDCs, as demonstrated by full competition with anti-CXCR4 antibodies. Binding of ^125^I-CXCL12 was completely inhibited by unlabelled CXCL12 but also partially by unlabelled chemerin, demonstrating cross-competition by the ChemR23 ligand (Figure 6 B). We next performed competition experiments on cells prepared from ChemR23^−/−^ mice and showed that chemerin did not compete for ^125^I-CXCL12 binding, indicating that the absence of ChemR23 completely abolished the cross-inhibition by ChemR23-specific ligands (Figure 6 C). Despite expression of ChemR23 detectable by FACS at the cell surface (Figure 6 A), only weak chemerin binding was measured on wild type BMDCs (Figure S3). This weak signal is most likely due to the relatively low expression level of ChemR23 in leukocytes combined to the poor stability of our peptidic tracer in biological media. Indeed, even on CHO-K1 cells overexpressing ChemR23, the binding assay is tedious and the specific window rather small (Figure S4). We did not investigate ChemR23/CCR7 binding cooperativity on BMDCs, as ChemR23 is inactivated and downregulated in parallel to CCR7 induction during maturation of dendritic cells [23]. The presence of both ChemR23 and CCR7 is therefore transient on these cells and difficult to study.

## Discussion

The migration of leukocytes from the bloodstream towards sites of infection or inflammation is dependent on the gradient of chemotactic factors derived from pathogens or host tissues. A growing number of chemoattractant molecules involved in this recruitment have been identified. These include complement components (C5a, C3a), bacterial N-formylated peptides, lipid metabolites such as leukotriene B4, and chemokines [28]–[30]. All these molecules bind to receptors belonging to the G protein-coupled receptor family and trigger various cellular responses including chemotaxis, activation of integrins, release of proteolytic enzymes and ROS production. Chemerin, a 137-aa protein sharing structural similarities with cathelicidin precursors, cystatins and kininogens, but unrelated to chemokines, was identified in our laboratory as a new chemoattractant factor for leukocytes through its binding to the receptor ChemR23 [20]; [23]. ChemR23 is considered as a potential target for drug development in the frame of inflammatory and metabolic disorders, but little is known about its organization at the surface of immune cells and how this organization influences its function and the function of other receptors. Heteromerization is known to influence pharmacological properties of several GPCRs. In line with this new concept, we previously showed that heteromerization of some chemokine receptors is associated with negative binding cooperativity and impacts on the properties of some small molecule antagonists [4]; [7]–[9]. In this study, we showed that ChemR23 forms homomers as demonstrated previously for chemokine receptors. Our data also indicate that ChemR23 forms heteromers with the chemokine receptors CCR7 and CXCR4. However, the relatively low BRET signals detected for heteromers might reflect a weaker interactions between heterologous receptors. It is therefore not possible to conclude definitely from these data that ChemR23 forms heteromers with chemokine receptors under physiological expression levels.

Following co-expression of ChemR23 with CXCR4 or CCR7 in CHO-K1 cells, we observed negative binding cooperativity across the binding pockets of the receptor pairs, similarly to what we reported previously for chemokine receptor heteromers. Agonists of one receptor were able to compete for the binding of specific tracers to other receptors only on cells expressing pairs of receptors. The pharmacological consequence of ChemR23/CXCR4 co-expression was also demonstrated on mouse BMDCs expressing the two receptors endogenously. Importantly, the cross-inhibitory effect exerted by chemerin was specifically abrogated for cells derived from ChemR23 KO mice, demonstrating that ChemR23 expression at the cell surface is mandatory for the negative cooperative effects of chemerin to occur. Collectively, these data demonstrate the relevance of ChemR23/CXCR4 interactions in primary leukocytes and show that negative binding cooperativity can take place across receptors binding structurally unrelated proteins. This negative cooperativity might be linked to receptor heteromerization as showed previously for chemokine receptor pairs, although a contribution of downstream signaling events, such as G protein recruitment, cannot be excluded. Similarly to what was reported for chemokine receptors, we did not detect any synergistic signaling between ChemR23 and chemokine receptors as measured by calcium mobilization. This result is not surprising as ChemR23 signals through activation of G_i/o_ proteins as do chemokine receptors (20). Finally, we also showed that ChemR23 and chemokine receptors do not seem to co-internalize upon ligand stimulation. This observation might reflect a weak interaction between ChemR23 and chemokine receptors, as suggested by the relatively low signal in our BRET assay. On the other hand, absence of co-internalization was also reported previously for chemokine receptor heteromers (8). No definitive conclusion can be made concerning the apparent discrepancy between oligomerization and independent internalization. Additional experiments exploring the dynamics of receptor association and dissociation will be required to address this matter in more details.

Given our results, it will be interesting to investigate whether binding cooperativity occurs between chemokine receptors and other chemoattractant receptors expressed on leukocytes. Heteromerization of C5aR and the chemokine receptor CCR5 was previously reported [31]. The direct consequences of such interaction on binding properties were not investigated, but stimulation of cells co-expressing CCR5 and C5aR with C5a was associated to cross-phosphorylation and cross-desensitization of CCR5. Interestingly, the authors proposed a model in which the cross-regulation depends on the heteromeric assembly of CCR5 and C5a. Down-modulation of chemokine receptors by C5a, formyl peptides and non-chemoattractant molecules has been abundantly reported in the literature [32]–[34]. However, most of these studies were performed before the emergence of the GPCR oligomerization concept. Further studies will certainly be required to investigate in more details heteromerization of chemoattractant receptors with chemokine receptors and whether this interaction participates to their cross-regulation.

We also previously showed that the CXCR4-specific antagonist AMD3100 inhibits the function of CCR2 and CCR5 as the result of their heteromerization with CXCR4 [7]; [9]. In contrast to these previous observations, we showed here that AMD3100 does not cross-inhibit chemerin binding in cells co-expressing ChemR23 and CXCR4. Cross-inhibition by a small molecule like AMD3100 depends thus on the nature of the receptor with which CXCR4 is co-expressed. The reason for such selectivity is not known for sure. The absence of cross-inhibition by AMD3100 is not due to a lack of cooperativity, since the CXCR4-specific agonist CXCL12 competed efficiently for the binding of chemerin in cells expressing ChemR23 and CXCR4. One might postulate that only some heteromeric conformations accommodate conformational changes promoted by AMD3100. Another related hypothesis is that conformational changes induced by agonists and antagonists could propagate through distinct interfaces and/or molecular mechanisms in CXCR4/ChemR23 and other CXCR4 heteromers. Finally, we cannot formally exclude that ChemR23 has a weak propensity to interact with CXCR4 in physiological conditions and that AMD3100, in contrast to CXCL12, does not trigger the signaling event leading to ChemR23 cross-regulation. Identification of key structural elements involved in the cross-inhibition exerted by specific ligands will certainly help us to understand the molecular basis of negative binding cooperativity.

The heterodimerization of GPCRs and negative binding cooperativity between heteromer units has a number of consequences on the use receptors as therapeutic targets. Multiple receptors might be for instance targeted by molecules acting on a common heteromerization unit. On the other hand, the fact that a small molecule antagonist may affect the functional properties of receptors on which these molecules do not bind has important consequences in terms of potential side effects. A clear understanding of the dimerization properties of the target, and its functional consequences in the relevant cell types, will be required in the future for the design of new therapeutic drugs.

## Supporting Information

Figure S1 **A. Homo- and heteromerization of GABA receptor as measured by BRET.** HEK293T cells were transfected with a constant amount of the GABAbR1-h*R*Luc construct and increasing amounts of the GABAbR2-EYFP (▪), ChemR23-EYFP (•), CXCR4-EYFP (○) or CCR7-EYFP (□) constructs. The BRET signal was recorded 5 minutes after addition of coelenterazine H. All data points were performed in triplicate (error bars indicate S.E.M.). **B. Cell surface expression of receptors.** HEK293T cells were transfected with HA-ChemR23 only (A) or with HA-ChemR23 and Flag-ChemR23 (B), CXCR4 (C), CCR7 (D) or Rho-tagged TSHR (E) used as competitors as shown in Figure 2B. The expression level of each receptor was measured by FACS by using specific antibodies for receptors or tags (open histograms) and isotype monoclonals as controls (filled histograms).
(TIF)

Figure S2 **Characterization of CHO-K1 cells expressing CXCR4 + ChemR23 or CCR7+ChemR23.** Cells expressing CXCR4 + ChemR23 or CCR7 + ChemR23 were incubated with increasing concentrations of ^125^I-CXCL12 (A, B), ^125^I-CCL19 (D, E) or ^125^I-[145–157]-chemerin (C and F) and total binding (▪) was measured. After one hour incubation, unbound tracers were separated by filtration and filters washed twice before counting. Non-specific binding was determined in the presence of a 100-fold excess of unlabeled CXCL12, CCL19 or chemerin (□), and the specific binding (•) was calculated as the difference. One representative experiment out of 3 is shown.
(TIF)

Figure S3 **Internalization of ChemR23 and chemokine receptors.** [**A–B**] Cells co-expressing ChemR23 and CXCR4 were left untreated (NS) or stimulated 90 minutes with 100 nM CXCL12 or chemerin. Surface-bound chemerin was removed by an acid wash step and cell surface expression of ChemR23 and CXCR4 was estimated by FACS. [**C–D**] Cells co-expressing ChemR23 and CCR7 were left untreated (NS) or stimulated 90 minutes with 100 nM CCL19 or chemerin. Surface-bound chemokines were removed by an acid wash step and cell surface expression of ChemR23 and CCR7 was estimated by FACS. The data were normalized for the expression of receptor in absence of stimulation (100%). Statistical significance as compared to the 100% values was tested by two-way analysis of variance followed by Tukey's test (***, *P*<0.001; **, *P<0.01*; *, *P<0.1*). All points were run in duplicated and the displayed data are mean of three independent experiments (error bars indicate S.E.M.).
(TIF)

Figure S4 **Competition binding assays on CHO-K1 and BMDCs.** Competition binding assays were performed on CHO-K1 cells expressing ChemR23 (**A**) or BMDCs generated from wild-type or ChemR23^−/−^ mice (**B**) by using 0.2 nM ^125^I-[145–157]-chemerin as tracer. After one hour incubation, unbound tracers were separated by filtration and filters washed twice before counting. The data represent binding in the absence of competitor (Black bars) and nonspecific binding in the presence of 300 nM of chemerin (White bars). All points were run in triplicates (error bars indicate S.E.M.).
(TIF)
