# Supplementary figures and images for: Consequences of ChemR23 Heteromerization with the Chemokine Receptors CXCR4 and CCR7
Source: PLoS One. 2013 Feb 28;8(2):e58075. doi: 10.1371/journal.pone.0058075 (PMC3585228; doi:10.1371/journal.pone.0058075)

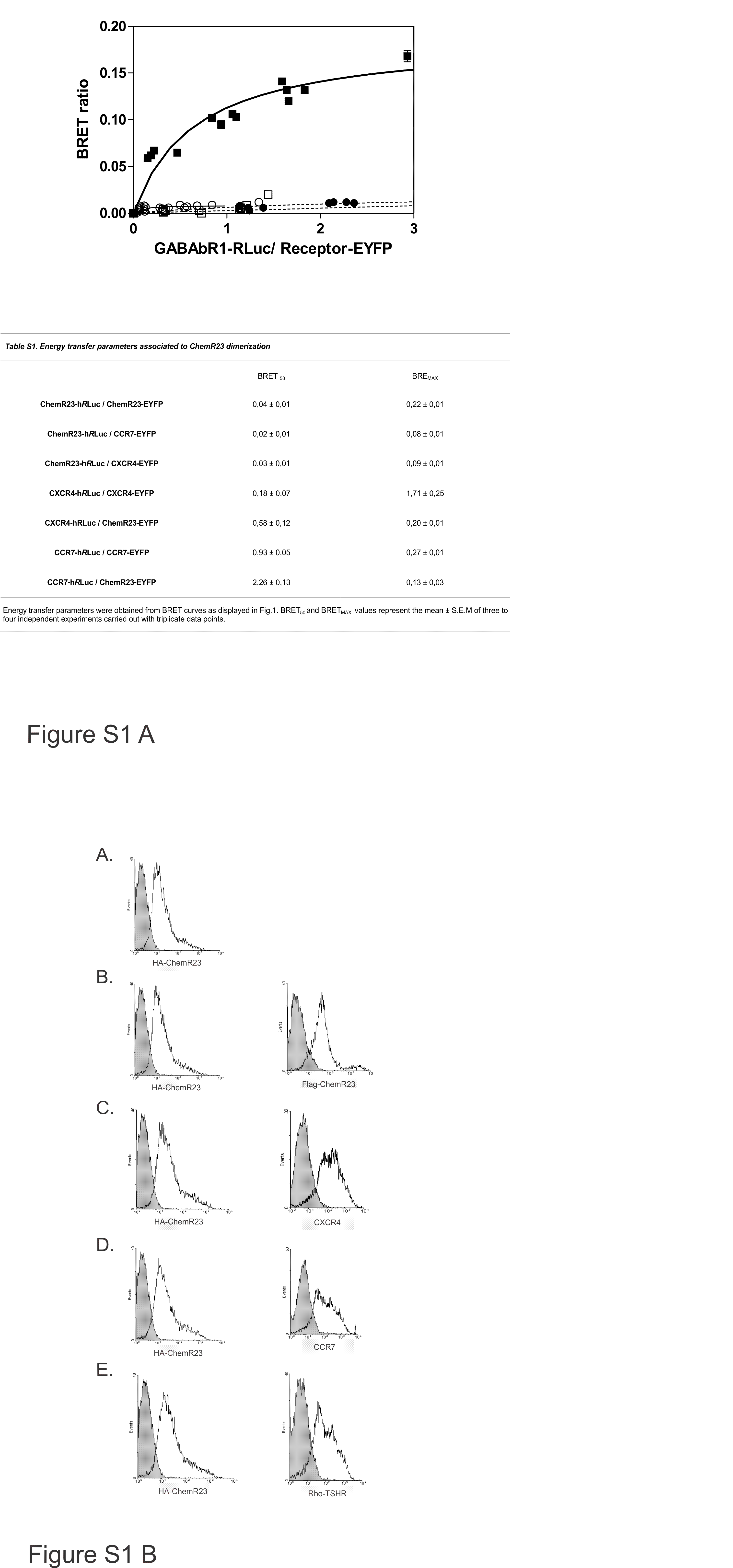

Supplement: Figure S1 — A. Homo- and heteromerization of GABA receptor as measured by BRET. HEK293T cells were transfected with a constant amount of the GABAbR1-hRLuc construct and increasing amounts of the GABAbR2-EYFP (▪), ChemR23-EYFP (•), CXCR4-EYFP (○) or CCR7-EYFP (□) constructs. The BRET signal was recorded 5 minutes after addition of coelenterazine H. All data points were performed in triplicate (error bars indicate S.E.M.). B. Cell surface expression of receptors. HEK293T cells were transfected with HA-ChemR23 only (A) or with HA-ChemR23 and Flag-ChemR23 (B), CXCR4 (C), CCR7 (D) or Rho-tagged TSHR (E) used as competitors as shown in Figure 2B. The expression level of each receptor was measured by FACS by using specific antibodies for receptors or tags (open histograms) and isotype monoclonals as controls (filled histograms). (TIF) [file pone.0058075.s001.tif]

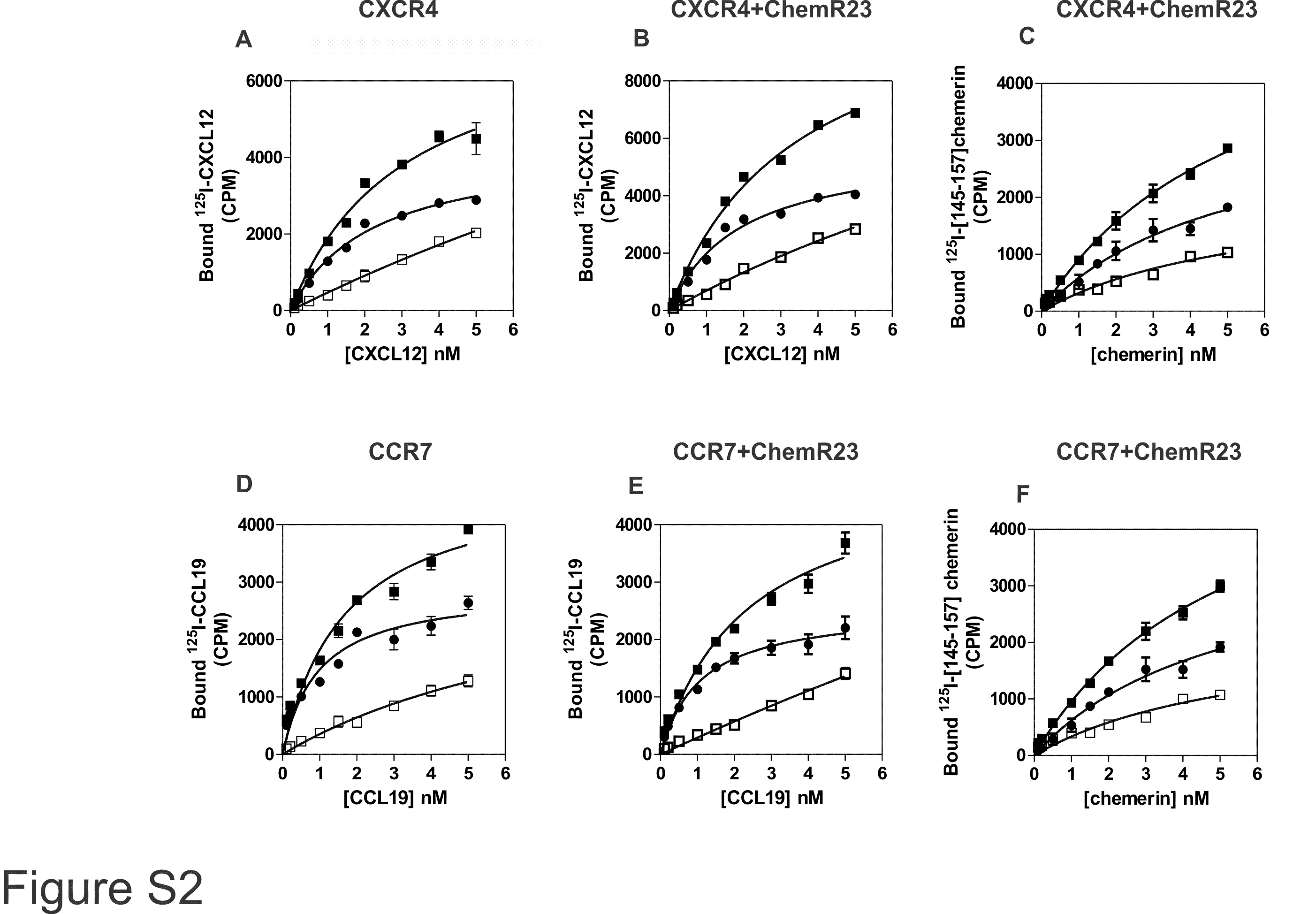

Supplement: Figure S2 — Characterization of CHO-K1 cells expressing CXCR4 + ChemR23 or CCR7+ChemR23. Cells expressing CXCR4 + ChemR23 or CCR7 + ChemR23 were incubated with increasing concentrations of 125I-CXCL12 (A, B), 125I-CCL19 (D, E) or 125I-[145–157]-chemerin (C and F) and total binding (▪) was measured. After one hour incubation, unbound tracers were separated by filtration and filters washed twice before counting. Non-specific binding was determined in the presence of a 100-fold excess of unlabeled CXCL12, CCL19 or chemerin (□), and the specific binding (•) was calculated as the difference. One representative experiment out of 3 is shown. (TIF) [file pone.0058075.s002.tif]

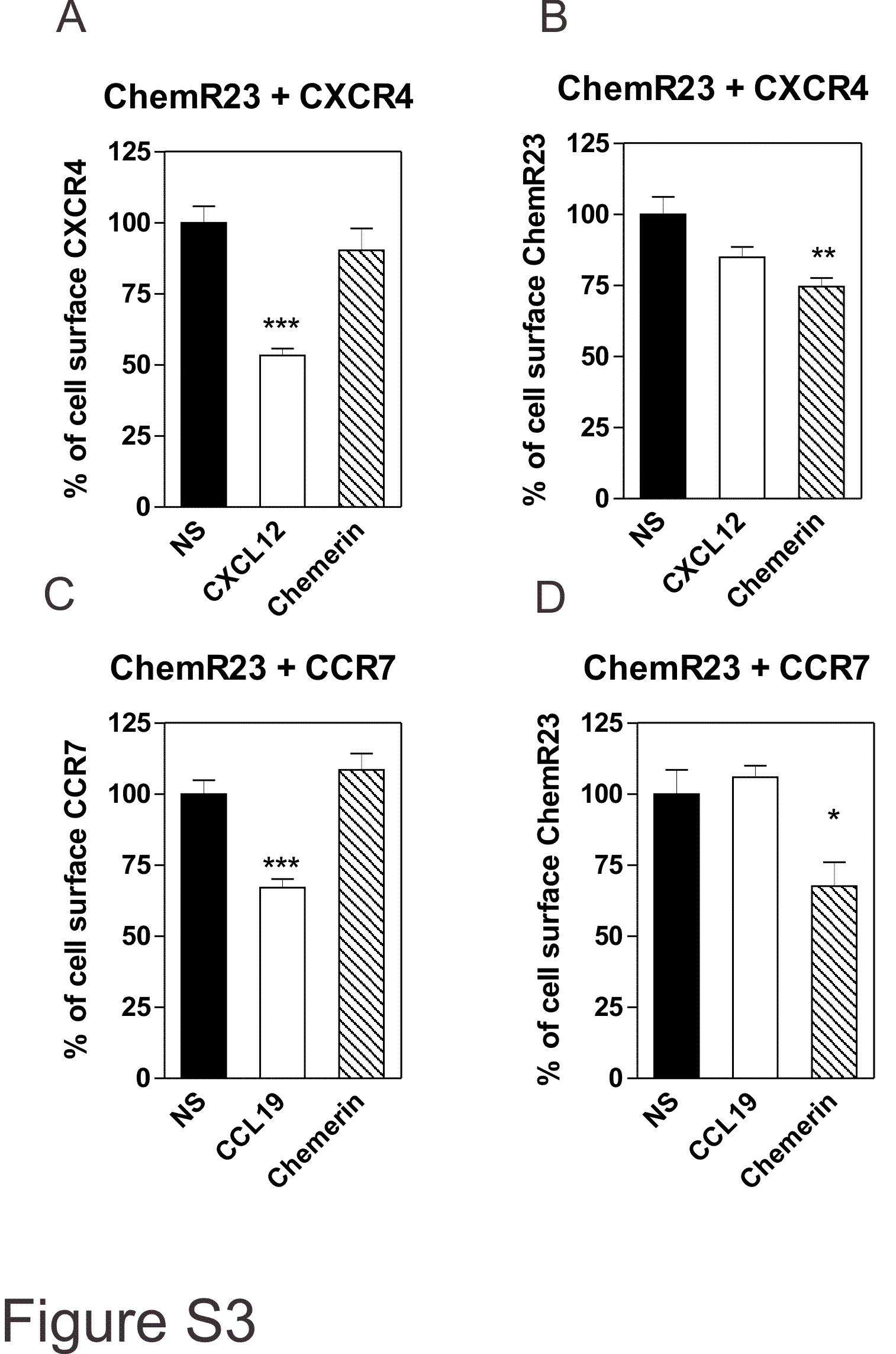

Supplement: Figure S3 — Internalization of ChemR23 and chemokine receptors. [A–B] Cells co-expressing ChemR23 and CXCR4 were left untreated (NS) or stimulated 90 minutes with 100 nM CXCL12 or chemerin. Surface-bound chemerin was removed by an acid wash step and cell surface expression of ChemR23 and CXCR4 was estimated by FACS. [C–D] Cells co-expressing ChemR23 and CCR7 were left untreated (NS) or stimulated 90 minutes with 100 nM CCL19 or chemerin. Surface-bound chemokines were removed by an acid wash step and cell surface expression of ChemR23 and CCR7 was estimated by FACS. The data were normalized for the expression of receptor in absence of stimulation (100%). Statistical significance as compared to the 100% values was tested by two-way analysis of variance followed by Tukey's test (***, P<0.001; **, P<0.01; *, P<0.1). All points were run in duplicated and the displayed data are mean of three independent experiments (error bars indicate S.E.M.). (TIF) [file pone.0058075.s003.tif]

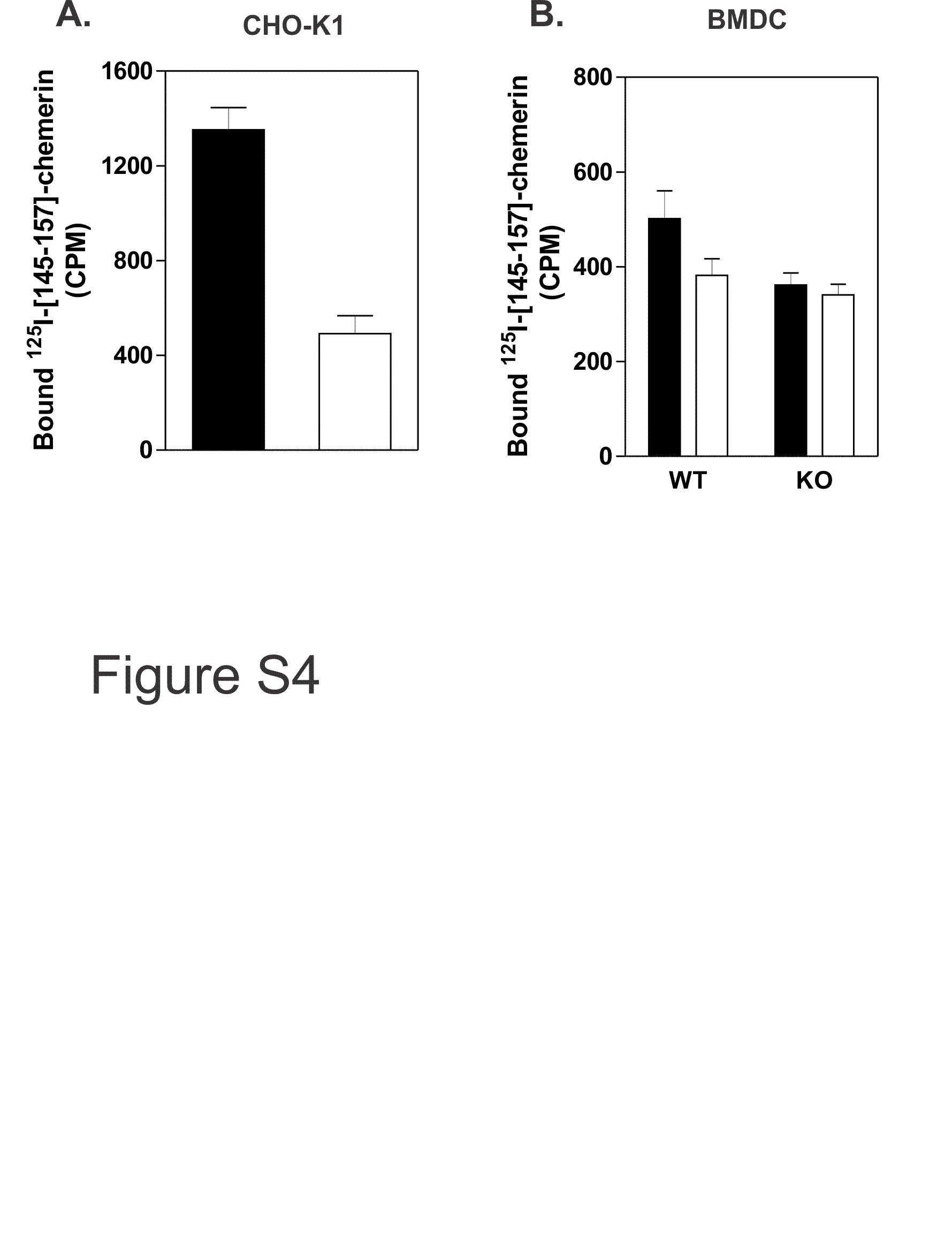

Supplement: Figure S4 — Competition binding assays on CHO-K1 and BMDCs. Competition binding assays were performed on CHO-K1 cells expressing ChemR23 (A) or BMDCs generated from wild-type or ChemR23−/− mice (B) by using 0.2 nM 125I-[145–157]-chemerin as tracer. After one hour incubation, unbound tracers were separated by filtration and filters washed twice before counting. The data represent binding in the absence of competitor (Black bars) and nonspecific binding in the presence of 300 nM of chemerin (White bars). All points were run in triplicates (error bars indicate S.E.M.). (TIF) [file pone.0058075.s004.tif]
